# Mechanism and capacities of reducing ecological cost through rice–duck cultivation

**DOI:** 10.1002/jsfa.6223

**Published:** 2013-06-27

**Authors:** Pan Long, Huang Huang, Xiaolan Liao, Zhiqiang Fu, Huabin Zheng, Aiwu Chen, Can Chen

**Affiliations:** a College of Agronomy, Hunan Agricultural UniversityChangsha, 410128, China; b College of Agronomy and Biotech, China Agricultural UniversityBeijing, 100193, China; c Key Laboratory of Multi-cropping Cultivation and Farming System (Ministry of Agriculture), Hunan Agricultural UniversityChangsha, 410128, China; d College of Biosafety Science and Technology, Hunan Agricultural UniversityChangsha, 410128, China; e Agricultural Bureau of Ningxiang County, Soil and Fertilizer Workstation410128, China

**Keywords:** reduce, ecological cost, rice–duck cultivation, economic benefit

## Abstract

**BACKGROUND:** **Rice–duck cultivation is the essence of Chinese traditional agriculture. A scientific assessment of the mechanism and its capacity is of theoretical significance and practical value in improving modern agricultural technology**.

**RESULTS:** **The duck’s secretions, excreta and their treading, pecking and predation decrease the occurrence of plant diseases, pests and weeds, enrich species diversity and improve the field environment. The rice–duck intergrowth system effectively prevents rice planthoppers and rice leafhoppers. The control effects can be up to 98.47% and 100% respectively; it also has effects on the control of *Chilo suppressalis*, *Tryporyza incertulas* and the rice leafrollers. Notable control results are found on sheath blight, while the effects on other diseases are about 50%. Harm from weeds is placed under primary control; prevention of weeds is sequenced by broadleaf weeds > sedge weeds > Gramineae weeds. Contents of soil organic matter, N, P and K are improved by the system; nutrient utilization is accelerated, resulting in decreased fertilizer application. Greenhouse gas emissions are reduced by 1–2% and duck fodder is saved in this system. There is also an obvious economic benefit**.

**CONCLUSION:** **Compared to conventional rice cultivation, rice–duck cultivation shows great benefits to ecologic cost and economic income. © 2013 The Authors. Journal of the Science of Food and Agriculture published by John Wiley & Sons Ltd on behalf of Society of Chemical Industry**.

## INTRODUCTION

The paddy ecosystem does not only have the function of grain production, but also has an important coordinative effect upon the ecological environment. Intensive rice production ensures continuous increase of grain yield through massive application of fertilizers and pesticides, but at the same time results in problems concerning the ecological security, environmental health and food safety of rice.^1^ In recent years, due to environmental pollution and the development of environmentally friendly agriculture, rice–duck cultivation has become popular in the Asia-Pacific region,^2^–^3^ which improves the ecological environment and plays an important role in reducing the impact of conventional rice farming on the environment and ecological cost of rice production.^4^

In the rice–duck cultivation system, rice is central though duck is a crucial component. Generally, rice has to be transplanted for 1 week or be direct seeded for 20 days before the ducks join the field. Twenty-day ducks are best, as they are not scared of water and are not able to cause large-area damage to rice plants. Thanks to similar needs for the growth environment, they each promote the growth of the other. Being omnivorous, active and agile, ducks feed on weeds, dead leaves and pests in the field, during which process they stir up the water and soil and fertilize the field so that rice is stimulated to grow, soil nutrients are increased and the need for application of fertilizers and pesticides is lowered.^5^–^6^ Meanwhile, the paddy plants offer a shady and hidden shelter for ducks and the field provides various animals, plants and plankton to feed the ducks; consequently the quality of duck meat improves.^7^–^8^ A number of research studies have shown that, based on farming with less or no fertilizers and pesticides, rice–duck cultivation can boost the growth of rice,^9^ improve soil properties,^4^–^10^ prevent plant diseases and pests,^11^–^14^ lessen the emission of methane^15^ and lower the quantity of duck feeds.^8^ It is safe to say that the rice–duck cultivation is of significant ecological and economic benefits.^16^–^17^

Ducks can be kept in the field until the rice harvest so as to achieve the best ecological benefits. 150–225 ducks are needed per hectare and should be introduced as early as possible.^18^ In this way, the symbiotic time is prolonged to the maximum and field resources, e.g. insects, weeds and leaves, can be made full use of. In order to achieve the best economic benefits, the key point is to maximize the density of ducks and shorten the pasture time appropriately. A suitable number for each hectare is 750; ducks should be driven out in the early heading stage, after which they are kept in captivity to grow rapidly and appear on the market as soon as possible. For this method, additional fodder is required to promote duck growth. In both methods, ducks play the role of cultivators and weeders, and control the occurrence of sheath blight, rice planthoppers, rice leafhoppers, and many other factors.^19^ Thus fewer pesticides, herbicides and fertilizers are used. However, the flowering and grain filling stage is a sensitive time for rice yield formation, and large ducks may injure the plants. To solve this problem, large ducks are replaced by smaller ducks younger than 50 days for fields with weeds, planthoppers and leafhoppers, for prevention and control, because they are undersized and cannot touch the rice spikes; for fields with no weeds or pests, there is no longer a need for ducks.

Previous studies mainly focused on particular ecological or economic benefits of rice–duck cultivation, and so far there have been no further analyses and summaries of the findings. This research, however, makes comparative studies on the ecological and economic benefits between the rice–duck cultivation system and conventional rice planting and probes the mechanism of such benefits of the rice–duck complex ecosystem. It analyses and quantifies the capacities of the system to prevent and control plant diseases, pests and weeds and to reduce greenhouse gas emission and duck feeds usage, and discusses the principles and mechanism of reducing the ecological cost. It is hoped to provide theoretical support for the rice–duck cultivation system and practical guidance for further development of the system.

## ECOLOGICAL COST AND SCOPE

The ecological cost, in its broadest sense, refers to the influence of human production and operation activities on the ecological environment^20^–^21^ or the pressure on the environment exerted by certain production activities.^22^ With regard to the rice cultivation system, ecological cost can be considered as the impact of the rice production in a specific area on the environment, including damage to and influence on water, atmosphere, soil and humans due to the application of pesticides, fertilizers, herbicides etc. The rice–duck intergrowth system introduces ducks into the conventional rice planting system, which changes the effects of the original system on the environment. A direct manifestation of the change is the reduction of fertilizers, pesticides, herbicides and feed application as a result of the ducks’ predation, weeding and intertillage fertilization. The improvement of rice quality and duck meat quality is another example.^23^ In a word, compared with the cost of conventional rice planting, the ecological cost is reduced by the rice–duck cultivation system and covers that of fertilizer, pesticide and herbicide application, greenhouse gas emission and duck feed. The changes in rice and duck meat quality are not within the scope of the research.

## MECHANISM AND CAPACITIES OF REDUCING PESTICIDE APPLICATION THROUGH RICE–DUCK CULTIVATION

### Mechanism of reducing pesticide application through rice–duck cultivation

Major pests in rice fields include rice planthoppers, *Chilo suppressalis*, rice leafhoppers and rice leafrollers.^17^ Yu *et al*. have found obvious predation of rice planthoppers and rice leafhoppers by ducks cultivated in the rice field, which becomes more significant with the growth of ducks.^9^ Certain control over stem borers and rice leafrollers are also revealed. Zhu *et al*. have proved that the rice–duck cultivation system can significantly prevent and control rice planthoppers and leafhoppers and can reduce damage from *Chilo suppressalis*, rice leafrollers and *Naranga aenescens* Moores to some extent.^24^ Although ducks sometimes prey on large spiders, the population density of the dominant species of spiders is still significantly higher than that in normal paddy fields since the new environment has facilitated the prolification of the pest’s natural enemies. Therefore, the system is again proved to be beneficial to the prevention and control of pests, which is in line with Zhou’s findings.^25^ Lin *et al*. have discovered that the daily grazing time of ducks also has an impact on the control effect of pests: the longer the time, the better were the results.^26^ Based on the simulation results of system modeling, Qin *et al*. have found that the role of natural enemies cannot be ignored in pest control through rice–duck intergrowth.^27^

All in all, there are two major approaches of pest prevention and control through duck feeding: (i) the direct approach, i.e. the ducks’ predation of pests and their eggs, which reduces the number of pests; (2) the indirect approach, i.e. disrupting the favorable living environment of pests and protecting the species of natural enemies with a different ecological environment in the paddy field. The introduction of ducks converts the ecological niches of the original system, leading to new food chains and food webs, and a different living environment for and abundance of species in the system. On one hand, activities of ducks influence the number of natural enemies such as spiders and *Trichogramma*; compared to conventional rice fields without duck grazing,^9^–^28^ the ratio of spiders to pests of the new system is higher and the parasitism rate of *Trichogramma* rises greatly.^24^ On the other hand, cluster activities of ducks improve the ecological environment of the rice field, destroy the living conditions of pests, accelerate the growth of rice and enhance its resistance.^29^ Possible mechanisms include the following: ducks change the activity routines of pests, increase the activity of insecticidal microbes, alter the palatability of pests’ prey and influence the occurrence time and growing space of pests; ducks’ activities stimulate the growth of roots, make leaves secrete more phenolic acids and increase the cuticula thickness, lignin content and leaf hardness.^30^ The physiological mechanism of how the rice–duck cultivation system prevents and controls pests will be a major area in future research as there have been very few in-depth studies until now.

### Capacities of reducing pesticides application through rice–duck cultivation

The rice–duck cultivation system has a notable control effect on pests in paddy fields but the results are inconsistent (Table1). Generally speaking, its effect on rice planthoppers and leafhoppers is higher than that on stem borers and rice leafrollers. The effect on rice planthoppers can be up to 98.47%, and on rice leafhoppers 100%. Therefore, the system can be a perfect substitution for chemical control and its effect can even be better than that of the latter.^24^ According to some research reports, its control effect on stem borers and rice leafrollers is not so significant. Zhu *et al*.,^24^ Deng and Pan,^28^ Yang *et al*.^31^ have found that the control effect on stem borers is not so good as that of chemical pesticides. Tojo *et al*. have discovered that a better control effect is found on rice leafhoppers and *Lissorhoptrus oryzophilus* Kuschel than on rice leafroller and that the system exerts no influence on the growth of spiders but increases their number.^32^ Teo *et al*. considered ducks to be a special biological control agent against golden apple snails, from which the control effect of ducks on *Ampullaria gigas* in south China can be inferred.^33^

**Table 1:** Control effects of rice–duck cultivation on pests

				Compared to control, pest density decrease ± (%)				
Ref.	Year	Season	Control setting	Rice planthopper	Rice leafhopper	*Chilo suppressalis* (*Tryporyza incertulas*)	Rice leafroller	Ratio of spiders to pest increase (%)
29	2008	E	CK	73.60	96.10	54.40	48.80	—
24	2001	E&L	CP	17.2—95.8	—	−15.63–7.32	46.67—63.64	30.48–1110.08
	2002	E&L	CP	61.8—94	—	9.38–12.5	33.30	126.89–1579.94
28	2005	L	CK	73.50	—	25.70	28.60	—
	2006	E	CK	—	—	50.40	50.00	—
	2006	L	CK	77.00	—	64.50	33.70	—
9	2001	M	CK	72.8—84.04	100.00	29.37–31.3	27.12–29.43	—
26	1999–2000	E&L	CK	81.62—98.32	75.0—93.45	83.33–95.04	72.73–100	—
31	2003	M	CK	≤72.84	—	≤57.3	≤19.4	Spider number decreases by 5.00
			CP	≤79.20	—	≤70	—	Spider number increases by 63.3
37	2003	L	CK	18.86—95.65	—	46.34 and 67.5	33.71 and 48.87	—
38	2004	M	CK	65.49	—	16.94–34.59	21.67–24.09	Increases by 18.74
39	2005	M	CK	—	—	19.20–46.20	50.00–66.70	—
34	—	NM	CK	75—98.47	—	85.61–88.76	72.59–87.96	Ratio increases by over 2 times
32	1999	NM	CK	—	≈80(375 ducks.ha^-1^) ≈45.5(195 ducks.ha^-1^)	—	−39.00 (375 ducks ha^−1^) −100 (195 ducks ha^−1^)	Has little influence on spiders

For the ‘Season’ column, E stands for early rice, L for late rice, M for middle rice, NM for not mentioned; for the ‘Control setting’ column, CK means rice cultivation with no pesticide and herbicide and without ducks, CP means conventional planting, i.e. rice cultivation with pesticide and herbicide but without ducks, RD means rice cultivation with ducks.

Conversely, other research studies have shown that the control effect of the rice–duck cultivation system on rice stem borers and leafrollers is up to 85% or more and the pest population is within the chemical control limit.^26^–^34^ One possible explanation for the difference is the rice varieties and duck types that researchers have chosen, as ducks of different types have disparate vertical ranges of activity. Generally speaking, smaller ducks have smaller vertical ranges of activity than larger ones; besides, with growing rice plants, the control effect of ducks on rice leafrollers and *Chilo suppressalis* on the upper part of rice plants decreases. In addition, the grazing time of ducks also exerts an influence upon the pest control effect. The best effect results when the frequent activity sessions of ducks coincide with the critical growth periods of larvae.^27^

The control effect of ducks on pests is subject to other factors. Qu holds that the rice–duck cultivation system can significantly decrease the base number of pests and mitigate the degree of injury.^35^ In a specific range, the effect becomes better with the increased density of ducks. Ye *et al*. have reached a similar conclusion.^18^ Lu *et al*. discovered a decreased control effect on pests and spider numbers with increased nitrogen fertilizer application.^36^

In general, the control effects of the system on rice planthoppers and rice leafhoppers are 60–99% and 75–100%, respectively. The effects on *Chilo suppressalis* and rice leafrollers are below 60%. Pesticides can be simply omitted or used once during the late period of rice growth.

## MECHANISM AND CAPACITIES OF REDUCING FUNGICIDE APPLICATION THROUGH RICE–DUCK CULTIVATION

### Mechanism of reducing fungicide application through rice–duck cultivation

Rice diseases arise from pathogenic bacteria invasion. There are over 240 types of diseases, of which rice blast, bacterial blight and sheath blight are the most widespread and hazardous. These three types are major epidemic diseases in China’s rice crops.^40^

Yu *et al*. has found a far lower disease index of sheath blight compared with the index in paddy fields without ducks.^9^ It is likely that ducks’ activities in fields bring a change in the original environment which is fit for mycelia reproduction and thus alleviates the spread and hazard of sheath blight. After some years studying the influence of rice–duck cultivation on rice sheath blight, Zhang *et al*. have discovered in duck droppings phenolic acids and organic matter containing mercaptan that inhibit sheath blight and extracts from the droppings antagonistic to bacterium A168.^41^–^42^ Zheng *et al*. have studied ducks feeding in no-tillage paddy fields and revealed that when ducks stir up the water in the fields the mud smeared on to rice stalks has a certain protective effect.^43^ Zhang *et al*. mud-capped the rice sheath and then inoculated *Rhizoctonia solani*.^44^ It was found that the disease incidence and severity index on rice plants were lower than that on control plants and plants with chemical prevention, which justifies the above-mentioned conclusion.

Based on the previous studies, the mechanisms of how rice–duck intergrowth affects pests mainly lie in the following aspects: (i) Prevention—ducks’ activities in fields improve air and light conditions, reduce relative humidity, maintain the stability of temperature and humidity, and thus help to create an environment against bacteria growth and reproduction; ducks, during their activities, smear mud on to rice stalks, protecting them against pathogenic bacteria; fertilizer application in the rice–duck cultivation system is decreased and ducks peck at ineffective tillering,^24^ so that the disease resistance of rice is enhanced. (2) Control—ducks’ pecking at some sclerotia cuts down origin of bacteria; ducks’ pecking destroys a few germinating hyphae which gradually shrink, stopping infection;^45^ an antimicrobial substance in duck droppings inhibits the growth and spread of bacteria. Besides, it is also likely that some chemical matter and microbes on ducks exert an influence on rice resistance and pathogenic bacteria^30^ but further research on this is needed.

### Capacity for reducing fungicide application through rice–duck cultivation

Loss due to sheath blight ranks top of all diseases and pests causing harm to rice plants.^45^ From Table2 it can be seen that sheath blight severity indices in different studies vary greatly but every system shows a notable control effect on the disease. In this aspect, no significant difference from conventional rice planting is shown and it is even better than the results of common chemical control, as has been revealed from studies by Zhu *et al*.,^24^ Gan *et al*.^7^ and Yu *et al*.^9^ Surveys by Ekurem^46^ and Yang *et al*.^31^ have also proved mitigated damage in the rice–duck cultivation system in comparison to conventional paddy fields.

**Table 2:** Comparison of control effects of rice–duck cultivation on sheath blight

Ref.	Year	Season	Incidence proportion of rice sheath blight (%)					
			CK	CP	RD
45	2003	M	45.13	19.63	18.67
	2003	L	49.1	15.01	34.11
9	2001	M	18.2–49.3	15.4–46.7	6.4–19.4
28	2006	E	0.23	0.07	0.08
	2006	L	0.29	0.08	0.08
24	2001	E	5.04 and 8.67	4.31 and 5.47	3.58 and 5.34
29	2008	E	—	—	21.1^a^
48	2000–2001	E&L	—	8.1 and 17.1^a^	6.3 and 11.8^a^
37	2003	M	0.66–58.64	—	0.27–15.25
7	2000-2001	E	—	30.7^b^	23.9^b^
		L	—	33.5^b^	26.5^b^
55	2003-2005	M	7.41 and 25.33	3.59 and 10.77	4.42 and 9.63
38	2004	M	0.72–25.62	0.34–7.34	0.56–16.25

For the ‘Season’ column, E stands for early rice, L for late rice, M for middle rice; for the ‘Control effect on sheath blight’ column, CK means rice cultivation with no pesticide and herbicide and without ducks, CP means conventional planting, i.e. rice cultivation with pesticide and herbicide but without ducks, RD means rice cultivation with ducks; lower-case letter ‘a’ represents the rate of disease plants and ‘b’ represents the occurrence rate.

The influence of the system on other diseases varies. Zhen *et al*. makes it clear that the integrated control effect of rice–duck intergrowth on rice blast reaches 57.02%;^38^ Shen *et al*. discovered that the system can evidently decrease the incidence of rice blast and large-scale rice–duck cultivation reduces the rate of diseased plants by 48.5%;^29^ Dai *et al*. reports that rice blast and bacterial blight of paddy fields in Yunnan are effectively controlled after rice–duck cultivation is implemented;^47^ Lu *et al*. also prove no difference between the rice–duck intergrowth region and chemical controlled region, both showing hardly any case of sheath blight or rice blast.^36^ However, Tong believes the system has no control effect on rice blast.^48^ One possible explanation is that ducks are not the direct agents exerting an impact upon rice blast but inhibit the occurrence and spread of the disease by changing the ecological environment. With no consistent activity area,^29^ if ducks are not well controlled during the critical period of disease occurrence, rice blast will become epidemic. Based on the above, the rice–duck cultivation system has a certain control effect on rice blast.

With regard to research on rice stripe disease, Zhen *et al*. concludes that the effect of the rice–duck intergrowth system can reach 79.44%—78.82% higher than the effect of conventional rice planting—and the best effect exceeds 94.01% if insect nets are covered at seeding stage and ducks are fed in fields afterwards;^49^ according to Sun *et al*., the control effect of the system on rice stripe disease is over 37.71%.^50^ Pandiarajan claims that the system cannot only improve rice yields and quality but also leads to fewer rice diseases.^51^

On the whole, the rice–duck cultivation system can effectively control rice sheath blight with a control effect of 60–100%, which is almost equivalent to the result of chemical control; it can control rice blast with a control effect of 50% or so, indicating needs for other measures based on field conditions; it can control rice stripe disease with an average control effect of more than 70%, implying basic control capacities over the disease. Therefore, chemical control over rice diseases is only recommended within a limited scope.

## MECHANISM AND CAPACITY FOR REDUCING HERBICIDE APPLICATION THROUGH RICE–DUCK CULTIVATION

### Mechanism of reducing herbicide application through rice–duck cultivation

Chemical weed control is common practice in agriculture at present but it results in terrible environmental pollution. A great many research studies have shown the evident control effect of rice–duck cultivation on the growth of weeds.^9^,^24^ The control is realized mainly in two ways: (i) direct effect, referring to ducks’ pecking at and treading on weeds; also, during ducks’ activities, they peck at weed seeds originally buried underground and this helps to decrease weed incidence in the following season; (2) indirect effect—when ducks stir up the water in the paddy field, the muddy water inhibits the germination and normal growth of weeds;^16^ the fields maintain water with a depth of 3–5 cm, impeding the germination and growth of weeds that are not resistant to a deep-water environment.^52^ Additionally, the chemical substance and droppings of ducks may also be inhibitive to weeds and the rice–duck cultivation system, while stimulating the growth of rice, at the same time suppressing the growth of weeds.

### Capacity for reducing herbicide application through rice–duck cultivation

The rice–duck cultivation system has a significant control effect on weed density in the rice field (Table3), but research results are inconsistent owing to the intervention of humans. With the prolonged grazing time and daily growth of ducks, more pests are consumed by ducks and their trampling abilities increase, meaning strengthened control over weeds; the herbicide effect of conventional weeds control methods, however, becomes weak over time, as does their control effect. According to research by Yu *et al*., after 10 days’ grazing the average weed control effect of the rice–duck cultivation system is 25.8% lower than the effect of herbicides, but after 40 days’ grazing the former is 91.5% above the latter—far beyond the control capacity in conventional rice planting.^9^ In comparison to conventional control methods, the rice–duck cultivation system prevails in terms of its control effect on weeds. Qu^35^ and Hou *et al*.^39^ have reached another conclusion: that is, the control effect on weeds is related to the number of ducks grazed. The greater the number of ducks, the lower is the density of weeds, which means a more significant control effect on weeds. The conclusion was verified by Li *et al*.^11^ and Liu *et al*.^45^ A grazing density of 225–300 ducks ha^−1^ will bring about better control effect of weeds than chemical herbicides.^18^ Wei *et al*. found through four years’ rice–duck cultivation that the weed density slowly decreases with every passing year.^52^ It fell by 99.82% after 4 years, which is significantly higher than that in the control region.

**Table 3:** Comparison of control effects of rice–duck cultivation on weeds

Ref.	Year	Season	Weeding effect of different symbiosis period (%)				Overall control effect (%)					
			RD ≦ 30d	CP	RD ≧ 40d	CP	RD	CP
24	2004	E	93.10	84.00	97.90	76.10	—	—
		L	88.00	76.00	97.70	72.70	—	—
		L	91.90	77.30	97.30	71.40	—	—
16	2000–2003	M	—	—	—	—	89.53	—
52	2002	M	—	—	—	—	95.60	—
28	2005	L	—	—	—	—	91.66	80.83
	2006	E	—	—	—	—	92.60	98.73
	2006	L	—	—	—	—	96.55	93.50
11	2003	L	88.00	77.00	96.40	55.70	—	—
37	2003	M	—	—	—	—	≧88.89	—
9	2001–2002	M	50.60	68.20	94.20	49.10	—	—
55	2003–2005	M	67.73	75.41	98.07	68.04	—	—
35	2009	M	55.6–85.2	86.7–79.2	61.2–89.0	68.40	—	—
31	2003	M	—	—	—	—	98.80	85.98
39	2005	M	41.6–86.4	25–93.2	56.4–91.1	42.8–76.3	—	—
53	2002	M	98.80	85.90	99.30	92.40	—	—
		L	98.50	82.40	99.20	93.50	—	—
38	2004	M	—	—	—	—	96.10	73.90
32	1999	NM	—	—	100.0^c^	0.0^c^	—	—

For the ‘Season’ column, E stands for early rice, L for late rice, M for middle rice, NM for not mentioned; for the ‘Control effect on sheath blight’ and ‘Overall control effect’ columns, CP means conventional planting, i.e. rice cultivation with pesticide and herbicide but without ducks, RD means rice cultivation with ducks; lower-case letter ‘c’ represents the control effect on *Monochoria vaginalis*.

Ducks feed on different weeds, so the control effect over weeds varies. There are over 200 kinds of weeds in the paddy fields of China, of which more than 20 kinds may lead to severe damage.^54^ According to Wei *et al*., the control effect of rice–duck intergrowth on broadleaf weeds is 97.1%, which is the highest, followed by effects on sedges and grasses with respective rates of 94.5% and 82.4%;^52^ the effect on dicotyledonous weeds are better than that on monocotyledonous weeds and the effect on broadleaf weeds is better than that on sedges and grasses, but the differences are not significant. Deng and Pan claim that the system has a better control effect on *Monochoria vaginalis* and *Rotala indica* and that the effect on barnyard grasses is relatively weak during the growing of early rice.^28^ Yu *et al*. prove that the control effects of the system on *Monochoria vaginalis*, *Marsilea quadrifolia* L. and *Rotala indica* can all reach 100%, while the control effects on *Sagittaria pygmaea*, sedges, barnyard grasses and *Ludwigia prostrata* are 62.17%, 41.94%, 61.27% and 42.55%, respectively.^55^

Qu’s study shows similar control effects of the system on broadleaf weeds and sedges, which are both better than the effect on grasses.^35^ Ma *et al*. demonstrate that the rate of control over weeds is more than 99.4% and only very few barnyard grasses are beyond control.^56^ In Yang’s survey,^57^ except for some barnyard grasses and fescues, the other weeds are all pecked at by ducks while in the conventional paddy fields fescues, *Monochoria vaginalis*, Potamogetonaceae, *Sagittaria trifolia* var. *ainensis* etc. are left alive. Tojo *et al*. believe that rice–duck cultivation can lessen weeds in the rice field and the control effect on *Monochoria vaginalis* can reach 100%.^32^

It is thus clear that the overall control effect of ducks on weeds is good—to be exact, 80% or so—and that the effect will become better over time of grazing; but the effect on barnyard grasses is weaker than that on other weeds due to the fact that barnyard grasses are usually mixed with rice plants as they grow among the latter and resemble the latter, so that ducks can hardly recognize them; another reason is that barnyard grasses are fast-growing and age quickly, whereas ducks dislike older weeds. Research results differ because the growth of weeds is not only subordinated to ducks, but also to water. For example, *Monochoria vaginalis*, barnyard grasses and sedges can grow in deeper water but *Ludwigia prostrata* can only live in shallow water. Generally speaking, as long as barnyard grasses are well controlled, the rice–duck cultivation system can control weeds without any application of herbicides.

## MECHANISM AND CAPACITY FOR REDUCING CHEMICAL FERTILIZER APPLICATION THROUGH RICE–DUCK CULTIVATION

### Mechanism of reducing chemical fertilizer application through rice–duck cultivation

Conventional rice fields give high yields depending on fertilizer application, which can meet the needs of rice plants for nutrients. However, overuse of fertilizers is bad for both environment protection and rice quality. The rice–duck complex ecosystem can promote the absorption of nutrients while improving soil nutrient conditions, structure and aeration conditions.^58^ Hayashi holds that rice–duck cultivation cannot only reduce pesticide application and fertilizer pollution, but also guarantees the safety of rice as food;^59^ Ahmed *et al*. agree that too much fertilizer will worsen the environment and threaten the safety of rice as food, while the rice–duck cultivation system has a positive effect against these problems.^60^ Previous research studies have concluded that the system is beneficial to the increase of soil N,^61^ and can minimize the loss of N and improve its application rate.^62^ The rice–duck cultivation system, with less use of pesticides and fertilizers, is capable of reducing environmental pollution. It can maintain a high level of soil fertility even when no fertilizer is applied.^63^

Based on these studies, a summary of how fertilizer application is reduced through rice–duck cultivation is as follows: (i) The decomposition of droppings and returning baits releases nutrients; fresh duck droppings are determined to contain 7.1 g kg^−1^ N, 3.6 g kg^−1^ P and 5.5 g.kg^−1^ K on average and are abundant in micro elements such as Fe, Mn, B and Ca.^5^ Given the assumption that the average fresh droppings of a duck weigh 0.14 kg a day and there are 225 ducks per hectare fed in paddy fields for 60 days, the statistics of paddy field input in every season are 13.42 kg N, 6.8 kg P and 10.4 kg K.^64^ (ii) Ducks’ pecking at algae and weeds in the rice field ensures decreased consumption of fertilizers. (iii) The abundant micro elements in duck droppings accelerate the decomposition and recycling of nutrients.^58^ (iv) The decomposition of organic materials in the rice–duck cultivation system and the disturbance of soil owing to ducks’ activities increase the oxidation–reduction potential of soil and boost the decomposition and effectiveness of nutrients.^5^ (v) Ducks’ activities in fields stimulate the growth of rice roots, which leads to a higher root:shoot ratio, a more vigorous root system and finally facilitates the absorption of nutrients by roots.^65^

### Capacity for reducing chemical fertilizer application through rice–duck cultivation

The effects of the rice–duck cultivation system on soil nutrients (Table4) consistently show that the system promotes organic matter content in the soil. This is because submerged soil is not well ventilated and thus not favorable for the decomposition of organic matter.^50^ Consequently, organic matter mounts. The contents of total nitrogen, total phosphorus and total potassium are all increased to various degrees, which is good for nutrient accumulation,^10^,^11^ but not all nutrients are increased. Shen *et al*. discovered that total N and available P on root surface and N and K in rhizosphere tend to fall and the soil ontology nutrient content is higher than that in conventional fields.^66^ This is because ducks’ activities in fields improve the ecological environment of the root surface and in the rhizosphere, enhance the absorption and utilization of nitrogen by rice plants and cause the falling content of total N. In general, rice–duck intergrowth boosts the emission of available N, available P and available K, the contents being higher than the conventional level but fluctuating over time. For instance, Zhang *et al*. have revealed that, without fertilizer application, the release of fertilizer efficiency in the rice–duck cultivation system is faster.^67^ In addition, different needs for nutrients in each growth period of rice is another influential factor.

**Table 4:** Comparison of effects of rice–duck cultivation on soil nutrients

Ref.	Year	The proportion of nutrients increasing amount (%)						
		OM	TN	AN	TP	AP	TK	AK
58	2000–2001 (E)	29.30	15.09	9.55	—	3.82	—	27.33
	2000–2001 (L)	11.30	2.96	5.94	—	9.70	—	23.36
64	2001	—	15.32	—	31.58	—	21.95	—
69	2005–2006	12.30	96.30	38.90	—	118.90	—	17.90
70	—	3.97	—	1.28	—	3.02	—	1.79
67	2002	6.26–21.77	—	7.11–81.4	—	−20.35–34.03	—	−5.26–40.59
66	2009 (root surface)	—	—6.15	21.74	5.88	−25.37	5.23	23.87
	(rhizosphere)	—	—7.24	—12.93	1.54	54.24	−11.78	−9.30
	(ontology)	—	4.61	5.78	4.76	13.5	2.22	12.22

For the ‘Year’ column, E stands for early rice, L for late rice; for the ‘Increase compared with conventional paddy fields’ column, OM stands for organic matter, TN for total nitrogen, AN for available nitrogen, TP for total phosphorus, AP for available phosphorus, TK for total potassium, and AK for available potassium.

Regarding the nutrient supply capabilities of the rice–duck cultivation system, Zhang *et al*. found that without fertilizer application organic matter, available phosphorus and available potassium in organic paddy soil can all meet the needs of rice in any part of its growth period.^63^ Jin *et al*. found that during 60 days’ cultivation the droppings of a duck weigh 10 kg, equivalent to 47 g N, 70 g P and 31 g K.^68^ If the duck density is 200 per hectare, the droppings can meet the nutrient needs of rice for N, P and K to guarantee its regular growth. Therefore, in the rice–duck cultivation system, no chemical fertilizers are needed for rice planting.

## MECHANISM AND CAPACITY FOR REDUCING GREENHOUSE EFFECT THROUGH RICE–DUCK CULTIVATION

### Mechanism for reducing greenhouse effect through rice–duck cultivation

Farmland is a major emission source of greenhouse gases CO_2_, CH_4_ and N_2_O.^71^ Taking up one third of the grain planting area, rice farming gives rise to large amounts of CO_2_, CH_4_ and N_2_O, which play an important role in the global greenhouse gas budget.^72^ Statistics show that rice fields emit 5–19% methane of the global total and 7–11% N_2_O of the national total.^73^ As a result, how to reduce greenhouse gas emission of paddy fields is a major concern in current studies.

The emission of different greenhouse gases is limited by the environment. CH_4_ is given off through three interactional processes, including the generation of CH_4_, its oxidation and emission into air.^73^ CO_2_/H_2_ and CH_3_COO^−^ are reduced by methanogens in the rice field under anaerobic conditions to generate methane.^74^ After the process of oxidation, the methane emission quantity accounts for 3–81% of the generated quantity.^75^–^76^ The oxidation of methane is closely connected with oxygen, controlled by microbes in soil and catalyzed by methane aerobic bacteria.^77^ There are three approaches to methane transport:^78^ (i) Methane is absorbed by rice plants and emitted into air through the ventilating tissue of plants; (ii) bubbles with CH_4_ are formed, rise to the surface of the water and then burst, giving off CH_4_; (iii) a little CH_4_ is spread and released from soil to water and further to the water–air interface, following the concentration gradient. N_2_O is an intermediate product in the processes of nitration and denitrification of nitrogenous compounds which involve microbes.^79^ Many studies have reported the tension between and the interaction in CH_4_ and N_2_O emission,^80^–^81^ which is mainly subject to the soil aeration conditions, soil temperature, soil moisture, soil texture, soil PH, soil oxidation–reduction potential, fertilizer application, irrigation, and so on. CO_2_ emission intensity depends on the content of organic matter in soil, their mineralization rates, the amount and activity of soil microorganism groups, respiration of animals and plants in soil, and so on. It is closely related to soil temperature and moisture.^79^

On the whole, rice–duck cultivation can reduce greenhouse gas emission. Zhan Ming *et al*. have found significant reduction of CH_4_ emission and increase of N_2_O emission through the system; but, as CH_4_ takes up 60% of the greenhouse gases in paddy fields, the global warming potential in the rice–duck cultivation system is significantly lower than that of conventional rice fields.^82^ Yuan *et al*., after undertaking a comprehensive analysis of 2 years’ experimental results, reached the same conclusion and found that the greenhouse effect is exactly 18.58% lower than that of conventional rice fields.^83^ Many other studies have also shown that the rice–duck cultivation system can significantly reduce the CH_4_ emission of paddy fields.^84^,^85^ Hence a lessened CH_4_ emission through the rice–duck cultivation system plays a critical role in the reduction of greenhouse gas emission.

The mechanism to reduce CH_4_ emission through rice–duck cultivation may be summarized thus: (i) ducks’ foraging and activities in paddy fields lead to better soil aeration and, as ducks feed on weeds and plankton, the latter will not consume as much dissolved oxygen as before, which accelerates the oxidation of methane but lowers its emission flux; (ii) the ineffective tillering of weeds and rice is pecked at, digested and then excreted by ducks, preventing them from decay and decomposition, during which processes methane may be given off;^85^–^87^ (iii) ducks’ disturbance of soil increases the chance of exposure to oxygen, destroys the reduction zone to inhibit the activity of methanobacteria, decreases the number of methanogens groups and, in the end, cuts down the emission of methane.^88^

### Capacity for reducing greenhouse effect through rice–duck cultivation

Table5 shows the results of the effect of the rice–duck cultivation system on greenhouse gas emission and greenhouse effect. It can be seen that the system reduces the emission of CH_4_ but increases the emission of N_2_O. Compared with conventional rice planting, the rice–duck cultivation system can cause the emission of CH_4_ to drop by 19.28–44.18%, far above the increase rate of N_2_O emission: 4.34–11.79%. The system promotes the growth of rice and the increased root:shoot ratio for efficient root respiration; duck droppings are rich in C and N, providing more substrate and energy for the respiration of microbes and facilitating soil respiration.^82^ Based on these findings, it is clear that the rice–duck cultivation system raises the emission of CO_2_. The rice–duck cultivation system as a whole can significantly accelerate the growth of rice plants and the fixation of CO_2_ on plants; duck droppings can add to the content of organic matter in soil and the fixation of C.^89^

**Table 5:** Comparison of effects of rice–duck cultivation on GHG emission and greenhouse effect

			CH_4_ emission (g m^−2^ per season)		N_2_O emission (g m^−2^ per season)		CO_2_ emission (g m^−2^ per season)		
Ref.	Year	Season	RD	CP	RD	CP	RD	CP	Reduction of greenhouse effect (%)
85	2002	L	12.2	17.79	—	—	—	—	31.60
83	2006	M	18.84	25.15	0.25	0.23	—	—	21.16
	2007	M	18.41	22.81	0.22	0.19	—	—	15.70
82	2007	M	18.41	22.81	0.20	0.18	273.66	245.73	7.20
62	2006	M	19.73	27.27	0.49	0.46	—	—	21.45
89	2006	M	19. 11	26. 71	0. 24	0. 23	−3441.95^d^	−3096.5^d^	60.83
15	2000–2001	L	10.11	17.05	—	—	—	—	40.70
	2001	E	5.52	9.89	—	—	—	—	44.22
84	2005	E&L	11.52	16.03	—	—	—	—	28.13
			13.99	21.35	—	—	—	—	34.47

For the ‘Season’ column, E stands for early rice, L for late rice, M for middle rice; CP means conventional planting, i.e. rice cultivation with pesticide and herbicide but without ducks, RD means rice cultivation with ducks; lower-case letter ‘d’ represents the quantity of CO_2_ fixed in soil. GWP is global warming potential.

The rice–duck cultivation system reduces the greenhouse effect by 7.2–60.83% of the amount in conventional fields. From the aspect of growing season, the late rice fields emit more methane than early rice fields, but the peak of emission occurs in mid-season rice fields. This is related to soil temperature; that is, when the soil temperature rises by 10 °C, the methane production rate becomes over four times the original amount.^77^ At the early stage of rice growth the temperature is relatively lower, so less methane is emitted; while at the middle and late stages, which are exactly the growing period of mid-season rice, higher soil temperature gives rise to more methane emission; at the middle and late growing stages of late rice, the temperature begins to fall and thus the methane emission diminishes again. A large number of research studies agree that good control of the rice–duck intergrowth period can reduce methane emission, lower temperature and alleviate the harmful effects of methane.^12^–^91^

## MECHANISM AND CAPACITY FOR REDUCING DUCK FEED APPLICATION THROUGH RICE–DUCK CULTIVATION

Ducks cannot grow without food. In the rice–duck cultivation system, ducks can live on feed offered by humans, as they can when kept in captivity, and foods from the paddy fields as well, such as weeds, insects, small tillering and old leaves on rice plants, as well as aquatic zooplankton and phytoplankton.^91^ In conclusion, the mechanism of how the rice–duck cultivation system reduces the duck feed application is as follows: (i) ducks can live on weeds and animals in the rice fields so they need fewer feeds; (ii) farmers will reduce the feed application to urge ducks to actively forage.^92^

Usually, ducks are provided with 70–80% of the feed of those kept in captivity, so that they will search for food actively.^93^ Huang *et al*. provide ducks with 60.00% of the feed for conventional breeding, and towards rice harvest the daily feed intake of each duck is 39.80% of the conventional amount and the feed conversion ratio falls by 26.69% 94. According to Lu *et al*.’s study, the amount of daily feed is lessened by 30.58% and the feed conversion ratio decreases by 3.30%. On the whole, the rice–duck cultivation system can save about 30% of feed.^95^

## ASSESSMENT OF ECONOMIC AND ECOLOGICAL BENEFITS OF RICE–DUCK CULTIVATION

Rice–duck cultivation has significant ecological effects but most assessments are made from the economic point of view. Zhang *et al*. calculated the net income from the rice–duck cultivation system in Zengcheng City of Guangdong Province in 2001 as $130.78 ha^−1^.^23^ If the price of rice from the system is 20% more than that of ordinary rice, the net income is US $323.52 per hectare more than with conventional cultivation. Huang *et al*. calculate that the net income of the intergrowth system in Hunan Province is US $647.04 per hectare more than that of the control field.^87^ Research on paddy fields in Hunan Province in 2008 has shown that large-scale rice–duck cultivation can earn $165.69 per hectare more net income than small-scale cultivation or US $221.71 per hectare more than conventional fields.^29^ Zhang *et al*. have made their calculation based on the price of organic rice and found out that even with reduced yields the rice–duck cultivation system can produce US $6478.195 per hectare more net income than conventional fields.^63^ This great difference derives from the fact that the price of organic rice is approximately eight times higher than that of ordinary rice.

Zu and Huang estimates, by means of the contingent valuation method, that the ecological service value of non-tillage rice–duck cultivation can reach US $3571.705 per hectare: 73.92% higher than conventional rice planting.^96^ Qin *et al*., using the ecological economics method,^64^ calculate that the ecological service value of the rice–duck intergrowth system is US $2928.635 per hectare.

Despite there being little assessment of the ecological service functions of the rice–duck cultivation system, it can still be easily figured out that the ecological service value of the system is more outstanding compared with its economic benefits; the significance of rice–duck intergrowth is more ecological than economic. For this reason, rice–duck cultivation is not only an approach to increase farmers’ income, but an effective way to develop sustainable agriculture.

## CONCLUSION AND PROSPECTS

The rice–duck cultivation system has better coordination performance and resistance capability in contrast to conventional rice planting, and it can notably decrease the ecological cost. It can be seen from the above discussion that the system can well control rice planthoppers and leafhoppers, and can prevent *Chilo suppressalis*, *Tryporyza incertulas* and rice leafrollers to such an extent that the ratio of rice plants to pests increases; it can well control the occurrence of sheath blight and prevent other diseases to some degree; its control effect on broadleaf weeds is best, followed by sedges and grasses in turn. Thus pesticide application is unnecessary on most occasions; it can improve the content of organic matter in soil, such as total nitrogen, total phosphorus and total potassium, ensuring better absorption of nutrients into the rice plants; it can reduce the greenhouse effect; and it can lower duck feed application.

In spite of the strong advantages over conventional rice planting, there are still key points to pay attention to in developing the rice–duck cultivation system:

1. Concerning rice production, the rice–duck cultivation system has significant ecological benefits but a low level of marketization. Rice products are usually sold at the price of ordinary rice, so farmers are under-motivated as the transmission between the ecological and economic benefits is not so desirable. This is where the crucial but difficult point lies in when developing the rice–duck cultivation system.
2. Concerning research, most studies are not sufficiently systematic, and mainly focus on a single aspect. The assessment is commonly made on a money standard rather than an environmental standard. This is the most important part in the rice–duck ecological system, though, which is certainly open for further improvement.
3. Although rice–duck cultivation can greatly reduce ecology cost, there are still some deficiencies in completely controlling the occurrence of pests, diseases and weeds. A complex ecological planting mode can be established by combining rice–duck cultivation with frequency trembler light trap or biogas engineering or other bio-engineering techniques to achieve better results.
